# Metabolic Disorders and Psoriasis: Exploring the Role of Nutritional Interventions

**DOI:** 10.3390/nu15183876

**Published:** 2023-09-06

**Authors:** Marco Cintoni, Marta Palombaro, Fabio Stefano Maramao, Pauline Raoul, Gabriele Egidi, Elena Leonardi, Luca Bianchi, Elena Campione, Emanuele Rinninella, Antonio Gasbarrini, Maria Cristina Mele

**Affiliations:** 1 UOC di Nutrizione Clinica, Dipartimento di Scienze Mediche e Chirurgiche Endocrino-Metaboliche, Fondazione Policlinico Universitario Agostino Gemelli IRCCS, Largo A. Gemelli 8, 00168 Rome, Italy; marco.cintoni@unicatt.it (M.C.); marta.palombaro@guest.policlinicogemelli.it (M.P.); gabriele.egidi@policlinicogemelli.it (G.E.); elena.leonardi03@icatt.it (E.L.); emanuele.rinninella@unicatt.it (E.R.); mariacristina.mele@unicatt.it (M.C.M.); 2 Centro di Ricerca e Formazione in Nutrizione Umana, Università Cattolica Del Sacro Cuore, Largo F. Vito 1, 00168 Rome, Italy; 3 UOSD di Dermatologia, Fondazione Policlinico Tor Vergata, Università degli Studi di Roma Tor Vergata, 00133 Rome, Italy; fsmaramao@gmail.com (F.S.M.); luca.bianchi@uniroma2.it (L.B.); elena.campione@uniroma2.it (E.C.); 4 Dipartimento di Medicina e Chirurgia Traslazionale, Università Cattolica Del Sacro Cuore, Largo F. Vito 1, 00168 Rome, Italy; antonio.gasbarrini@unicatt.it; 5 UOC di Medicina Interna e Gastroenterologia, Dipartimento di Scienze Mediche e Chirurgiche Endocrino-Metaboliche, Fondazione Policlinico Universitario Agostino Gemelli IRCCS, Largo A. Gemelli 8, 00168 Rome, Italy

**Keywords:** psoriasis, obesity, clinical nutrition, nutritional issues, nutritional supplementation

## Abstract

(1) Background: Psoriasis is a chronic autoimmune disease with a close relationship with metabolic diseases such as obesity, diabetes, and dyslipidemia. The aim of this review was to identify the relationship between psoriasis, metabolic diseases, and dietetic therapies. According to recent findings, there is a strong association between psoriasis and obesity as well as vitamin D and micronutrient deficiencies. (2) Methods: This review was conducted via PubMed, aiming to search for studies involving psoriasis linked with metabolic disorders or with nutritional treatments. (3) Results: Our review shows that a healthy lifestyle can positively influence the course of the disease. The maintaining of a proper body weight together with physical activity and good nutritional choices are associated with an improvement in psoriasis severity. A Mediterranean diet rich in fiber, vitamins, and polyphenols may indeed be a strategy for controlling psoriasis symptoms. The effectiveness of this diet lies not only in its anti-inflammatory power, but also in its ability to favorably influence the intestinal microbiota and counteract dysbiosis, which is a risk factor for many autoimmune diseases. (4) Conclusions: In synergy with standard therapy, the adoption of an appropriate diet can be recommended to improve the clinical expression of psoriasis and reduce the incidence of comorbidities.

## 1. Introduction

Psoriasis (PSO) is an immune-mediated, chronic inflammatory skin disease that affects about 100 million people worldwide [1]. In Europe, psoriasis affects approximately 2% of people [2], and the prevalence of the disease has also been found to be increasing [3]. It is characterized by erythematous scaly plaques, and it can involve the joints in the case of arthropathic psoriasis; severe disease is associated with significant impairment in physical and mental health because of its impact on daily activities. PSO can occur at any age, but people between the ages of 50 and 60 are more likely to experience it [4]. Obesity (OB) has become one of the leading health issues of the 21st century, with over one-quarter of the United Kingdom population now obese and similarly high OB levels in many other parts of the world [5]. In fact, observational evidence from epidemiological studies underlines strong associations between waist circumference and psoriasis risk, so central adiposity plays an important role in disease development. These data indicate that obesity is an important, potentially modifiable risk factor in the development of psoriasis that provides an opportunity for disease prevention [6]. OB could promote skin inflammation, but skin and joint disease can also lead to reduced participation in physical activity, resulting in weight gain. It is mandatory to understand cutaneous and systemic metabolic effects associated with obesity and psoriasis, which is an essential prerequisite to define treatment and prevention strategies for these prevalent public health issues.

In this article, we shed light on nutritional intervention and its role in treatment for PSO patients. The approach to these patients must be interdisciplinary and focus on both skin symptoms as well as recognizing and controlling comorbid conditions like cardiovascular, metabolic, nutritional, and sociopsychological issues. The aim of our work is to analyze how nutrition can help in the therapeutic management of this complex pathology.

## 2. Materials and Methods

This review was conducted on Pubmed via MedLine, from inception to November 2022, aiming to identify published studies exploring the role of metabolic disorder in PSO patients. We included for this review the following: (i) studies involving PSO patients; (ii) studies including information about OB, T2DM, celiac disease, and micronutrient deficiencies; (iii) diet, lifestyle changes, and micronutrient supplementation; (iv) all types of studies (including observational, prospective, and retrospective studies, case–control studies, cohort studies, narrative reviews, systematic reviews, and meta-analyses); and (v) studies written in English. All studies that did not fall into the previous criteria were excluded from the review process.

## 3. Psoriasis: Pathogenesis, Epidemiology, and Symptoms

Psoriasis is a chronic, immune-mediated skin disease that affects approximately 3% of the US population and an estimated 125 million people worldwide [7]. Plaque psoriasis is the most common variant, accounting for more than 80% of the psoriasis cases; other morphologic variants of psoriasis include guttate psoriasis, erythrodermic psoriasis, and pustular psoriasis as well as the involvement of the joints in the case of arthropathic psoriasis [8]. Plaque psoriasis most commonly occurs on the elbows, knees, and lumbosacral region, but it can affect any body surface. Plaque psoriasis can also affect areas such as the scalp/face (43–65%), nails (23–60%), palms/soles (12–26%), and genitalia (14–43%), which have been described as difficult-to-treat areas [9]. The diagnostic workup for psoriasis includes a family history of psoriatic diseases and a comprehensive skin and nail examination, which includes the evaluation of morphology and distribution of psoriasis lesions. In most patients, psoriasis can be diagnosed clinically. However, a skin biopsy may be required for cases in which the presentation is not typical [8]. PSO is recognized as a multifactorial disease that is triggered by a variety of genetic, immune, and environmental factors. Th cells are activated as part of the immune response, which additionally results in the release of pro-inflammatory cytokines like interleukin-1 beta (IL-1B), interleukin-17 (IL-17), interleukin-22 (IL-22), interleukin-23 (IL-23), and tumor necrosis factor alpha (TNF-a). These cytokines help explain why keratinocytes proliferate abnormally and differentiate insufficiently [10,11]. IL-23-mediated activation of the TH17 pathway is thought to be predominant. IL-23 signaling is mediated intracellularly via Tyk2-Jak2 and STAT3, which leads to transcription of key inflammatory mediators. These cytokines lead to downstream keratinocyte proliferation, increased expression of angiogenic mediators and endothelial adhesion molecules, and the infiltration of immune cells into lesioned skin [2]. Furthermore, over 60 susceptibility regions have been discovered through genetic studies, highlighting the role of genes involved in Th17 cell activation in the development of PSO [12].

Numerous environmental factors are acknowledged as “sets off” that may appear in people with a genetic predisposition, causing the disease to start or worsen. These sources of stress include exposure to cigarette smoke, infections, and certain medications (including beta-blockers, interferon, lithium, antimalarial medications, and nonsteroidal anti-inflammatory drugs) [13]. They also include excessive alcohol consumption and physical trauma such as injuries [14].

From 1990 to 2019, the global incidence of psoriasis increased from 3,653,236 to 4,622,594, with a percentage increase of 27%; this growth increases the importance of research toward this disease and different therapeutic approaches. While PSO onset can occur at any point in life, it typically begins between the ages of 20 and 30, peaks in incidence between the ages of 50 and 60, and is uncommon in children. An early PSO development (prior to the age of 15) is typically linked to a more severe form [15]. Males and females both experience PSO at the same rate [16].

The Psoriasis Area Severity Index score (PASI), one of the most common quantitative indices used to evaluate the severity and PSO extension, is a useful tool to assess psoriatic lesions based on the features of erythema, infiltration, and flaking [17]. Peeling of the skin (92%), itching of the skin (72%), erythema of the skin (69%), fatigue-asthenia (27%), inflammation of the skin (23%) plaque burning (20%), and skin bleeding (20%) are the most typical PSO symptoms; scaling was the most common symptom, which was experienced by 82% of patients, followed by itching (73%) and pain (32%). Increased psoriasis severity was associated with increased itching, pain, and scaling, resulting in reduced quality of life (QoL), worse work capacity, and difficulties in interpersonal relations [18]. It is therefore clear how the diseases and effects of PSO can worsen social relationships, physical disability, and work productivity, which results in reduced quality of life [18]. Treatment of these patients can start with topical corticosteroids and vitamin D derivatives in the case of mild psoriasis. Cyclosporine and methotrexate are options; however, long-term treatment with cyclosporin causes renal dysfunction, hypertension, and blood count abnormalities, and methotrexate is associated with hepatic and hematological toxicities. Furthermore, biologic drugs can be used to treat moderate to severe plaque psoriasis and they represent one of the most significant therapeutic advancements in the field of dermatology. The four main classes of biologics used to treat PSO are TNF inhibitors, IL-12/23 inhibitors, IL-17 inhibitors, and IL-23 inhibitors [8,9]. 

The purpose of our work is to analyze how nutrition can help in the therapeutic management of this complex pathology.

## 4. Emerging Associations and Mechanisms Linked with Psoriasis and Metabolic Disorders

PSO is associated with multiple non-communicable chronic diseases, such as Crohn’s disease, depression, cardiovascular disease, cancer, and metabolic syndrome [19]. According to clinical research, PSO can occur along with metabolic syndrome, type 2 diabetes mellitus, hypertension, hepatic steatosis, and non-alcoholic fatty liver disease [16,17,18]. Indeed, recently, a growing number of studies and recent meta-analyses have assessed a close relationship between PSO with metabolic disorders [20,21], including diabetes [22,23], obesity [24], vitamin deficiencies [25], and celiac disease [26]. Therefore, in this part, we highlight the established and emerging important mechanisms that link PSO with OB, diabetes, vitamin deficiencies, and celiac disease. Moreover, we dedicate a paragraph to microbiota gut dysbiosis, since several recent studies have identified gut-microbiota-dysbiosis-associated metabolic disorders as possible triggers or causes for PSO [27,28,29]. The main nutritional issues associated with PSO are summarized in Table 1.

### 4.1. Emerging Associations between Psoriasis and Obesity

OB is a chronic metabolic condition characterized by a rise in the amount and/or dimensions of adipocytes, which, as the threshold for the diagnosis, is a body mass index (BMI) of 30 kg/m^2^ or higher. Recent scientific research suggested a strong correlation between OB and PSO [15,24]; in fact, it has been assessed that environmental factors and excessive body weight are crucial factors that can increase clinical symptoms or even become triggers for the onset of the disease. The prevalence of obesity appears to be higher among individuals with PSO compared to the general population [40]. Some researchers contend that OB and OB develop after the onset of PSO, while others have discovered evidence that suggests OB and OB development are separate risk factors for the emergence of psoriasis [41,42,43]. However, because of the ongoing low-grade inflammation brought on by the release of pro-inflammatory cytokines from adipocytes, OB and overweight have been clinically identified as triggers for the manifestation of psoriatic pathology in genetically susceptible individuals [44]. There is a consensus among researchers that individuals who are overweight or obese may experience a worsening of psoriatic symptoms compared to those with a normal weight [30]. There is evidence that people with psoriasis (PSO) have a higher incidence of metabolic syndrome, according to a meta-analysis involving 15,939 PSO patients and 103,984 healthy controls. This study suggests periodically screening PSO patients for metabolic syndrome complications, such as hypertension, high triglyceride levels, low HDL cholesterol, elevated fasting blood glucose levels, and a large waist circumference [45]. 

Obesity is associated with a low-grade inflammatory status [33]. Adipose tissue is a source of pro-inflammatory cytokines such as IL-6, TNFα, and IL-8 [33]. Adipokines, proteins produced by adipose tissues, such as leptin, resistin, and adiponectin, are known to regulate lipid and glucose metabolism, inflammation, vascular homeostasis, and coagulation. The overproduction of adipokines such as leptin and resistin influences systemic inflammation. Adipokines have also been studied in psoriasis such as pro-inflammatory chemerin, lipocalin-2, and visfatin, as well as the anti-inflammatory adipokine omentin. A recent meta-analysis [46] found that lipocalin-2 and chemerin serum levels and resistin were increased in PSO patients compared to healthy controls. Moreover, unlike leptin and resistin, adiponectin is decreased in obese subjects compared to controls [47] as well as in PSO patients when compared with controls [48,49].

### 4.2. Emerging Associations between Psoriasis and Vitamin D

Several studies suggest an inverse relationship between vitamin D levels and body weight; in fact, hypovitaminosis D is the most frequent micronutrient deficiency among obese patients [32]. The adipose tissue represents the primary storage of vitamin D and, therefore, it is probable that the global epidemic of OB contributes to the high prevalence of hypovitaminosis D [32].

Notably, vitamin D has an important role in PSO. Sunlight exposure and vitamin D derivatives represent adjuvant treatments for an effective clinical response [50]. The importance of vitamin D primarily resides in its immunomodulatory capacity that counteracts PSO inflammation at the skin level. Vitamin D receptors (VDRs), but also the enzyme CYP27B1, which controls the local activation of 25(OH)D in 1,25(OH)2D, are expressed by activated T lymphocytes. The binding of these receptors determined the inhibition of the release of pro-inflammatory cytokines (IL-2, IFN-γ, IL-6, and IL-8) and lowered the cell-mediated immune response [51]. Secondly, Vitamin D has a direct action on keratinocytes. Epidermal cells express VDRs and Retinoid-related Orphan Receptors (RORs), which, in the presence of a high concentration of vitamin D, lead to inhibition of keratinocyte proliferation and increased cell differentiation [52]. Moreover, specific polymorphisms of VDR are associated with an increased severity of PSO as well as with the response to treatment with vitamin D derivatives [53]. Indeed, vitamin D levels tend to be lower in PSO patients than in healthy controls. Reduced plasma values seem to be related to increased duration and the most severe clinical disease presentation [54]. 

### 4.3. Emerging Associations between Psoriasis and Gut Microbiota

The community of bacteria and microbes living in the intestines is commonly identified as the gut microbiota. Through several mechanisms, it is essential for maintaining our general health. These include, among other things, assisting with digestion, promoting energy absorption, protecting against pathogens, producing the necessary nutrients, and regulating the immune system [55]. The emergence of autoimmune, inflammatory, and systemic diseases, including type 1 diabetes mellitus, rheumatoid arthritis, inflammatory bowel disease, and PSO, has been linked to dysbiosis, a condition in which the microbiota becomes unbalanced due to a reduction in microbial diversity. According to this viewpoint, since they decrease pathogenic bacteria, antibiotics, prebiotics, probiotics, and fecal transplantation may represent an option for treatment among patients with PSO [34]. Numerous studies have demonstrated in the literature that the gut microbiota of PSO patients and healthy controls differ significantly [35]. In general, they show a rise in the Firmicutes/Bacteroidetes ratio and a decline in the diversity of the microbiome [36,56,57]. BMI, exercise, lifestyle, and eating habits all have an impact on these changes, which are comparable to those seen in obese patients [58,59]. Increased gut permeability, which results in the translocation of bacterial components into the bloodstream, is linked to hyperglycemia. This is what leads to a low-grade inflammatory response, which has an undesirable effect on hormone production, glucose metabolism, and the development of several chronic disease conditions [60,61].

### 4.4. Emerging Associations between Psoriasis and Micronutrients Deficiencies

The deficiency of some micronutrients seems to be associated with psoriatic disease. Recent studies evidenced that it is common to find high blood levels of homocysteine in PSO patients, a condition that can be explained by a deficiency in folic acid and vitamin B12 [62,63]. Moreover, some authors reported that homocysteine levels directly correlate with PASI [62,64,65]. Among the causes of folic acid insufficiency, authors hypothesized the decreased intestinal absorption of micronutrients induced by systemic inflammation and the increased usage by skin epidermal cells [63,66]. 

Some antioxidants, including selenium, vitamin E, and beta-carotene, can be fundamental to correct the oxidative imbalance that supports the inflammatory state of PSO. The most in-depth research has been conducted on selenium. This nutrient is necessary for glutathione peroxidase to operate normally and can be detected in low amounts in PSO patients, especially in those who suffer from this condition for a long time [67].

Serum levels of vitamin E levels appeared to be lower in patients suffering from PSO (as well as other chronic inflammatory skin diseases) compared to healthy individuals [37]. Vitamin E acts as an antioxidant protecting cell membranes from reactive oxygen species [68]. Indeed, it is advisable to evaluate vitamin E status in PSO and to supplement it when needed [37]. Zinc has anti-inflammatory properties, and it is used with benefit as a supportive treatment for many inflammatory skin diseases [69]. 

### 4.5. Emerging Associations between Psoriasis and Diabetes

Several authors stressed the existence of a link between diabetes and PSO as they share some common pathogenic mechanisms [70,71]. Undoubtedly, inflammation is a hallmark of PSO and is a typical feature even in metabolic syndrome and diabetes [71]. Indeed, inflammation plays a pivotal role in the development of insulin resistance (IR) and IR has been frequently found in patients with PSO [19]. The excess of insulin binds to IGF receptors and stimulates the proliferation of keratinocytes and fibroblasts, thus representing a risk factor for the onset of psoriatic lesions [72]. Moreover, in the case of PSO, IGF levels in the bloodstream and on the skin can rise under the influence of the inflammatory cytokines released [72]. Adipokines are molecules that act as insulin sensitivity enhancers and anti-inflammatory cytokine inductors and are reduced in both PSO and diabetes [73]. 

Hyperglycemia is closely associated with psoriatic inflammation and some evidence supports a link between serum glycated hemoglobin (HbA1c) and PASI [74].

A similar immunological system and inflammatory signaling pathway suggested that people with PSO may also benefit from anti-inflammatory and anti-diabetic drugs [70]. For example, there is evidence that glucagon-like peptide 1 (GLP-1) receptor agonist, approved for type 2 diabetes, may also improve PSO [75,76]. GLP-1 analogs are effective in reducing symptoms of PSO in patients with type 2 diabetes independently of changes in weight and glycaemic control [76]. Experimental data support the hypothesis that this beneficial effect is related to the capacity of these drugs to interact with the innate immune system and reduce inflammatory cytokine production [70]. Similarly, pioglitazone and metformin showed interesting results in the control of skin lesions as they are insulin sensitizers with anti-inflammatory properties that can inhibit human keratinocyte overproliferation [77,78]. 

Interestingly, genetic analysis seems to support a linkage between PSO and diabetes. Specific susceptibility genes have been found in both type 1 diabetes and type 2 diabetes, and in PSO [71]. Mutations in genes or the dysregulation of microRNA (miRNA) expression is associated with a wide variety of human diseases, including diabetes and PSO [71]. Granata et al. describe a link between both diseases through miR-21, which is up-regulated in T-helper cells in psoriatic subjects and promotes the expression of genes involved in diabetes type 1 [73]. 

### 4.6. Emerging Associations between Psoriasis and Celiac Disease

Gluten is a protein naturally found in some grains, especially in wheat plants. It can also be added to food and processed food to add flavor or texture. The structure of gluten is composed of glutenin and gliadin, which are both responsible for most of the adverse health effects of gluten in genetically susceptible individuals [79]. In celiac disease (CD), gluten ingestion leads to a reversible inflammatory process in the small bowel mucosa with acute symptoms such as bloating, diarrhea, constipation, nausea, and vomiting. CD is an autoimmune-mediated disorder characterized by a specific genetic genotype (*HLA-DQ2* and *HLA-DQ8* genes) and autoantibodies (anti-tissue transglutaminase and anti-endomysial) triggered by consumption of gluten [80,81]. Non-celiac gluten sensitivity is characterized by similar symptoms seen in CD patients after consumption of gluten foods, but without an increase in antibodies, and intestinal damage has also grown in recent years in Western countries [82].

In the settings of PSO patients, pooled results from a recent meta-analysis highlighted that PSO could be significantly associated with the risk of developing CD [26]. Indeed, both PSO and CD patients may present an increase in levels of pro-inflammatory cytokines and similarities in terms of genetic susceptibility [83,84]. CD and even other autoimmune diseases such as type I diabetes mellitus and autoimmune thyroid disease might be associated with PSO development since *HLA-DR3*, *HLA-DQ2*, and *HLA-DQ8* are also involved in these diseases [38,85,86]. Furthermore, T helper type-1 (Th1) cells were activated in CD as well as PSO [87]. In PSO, the response of T helper 1 cells is associated with an increase in levels of IL-1 and IL-18, promoting the hyperproliferation of keratinocytes. Interestingly, in CD, dietary gluten consumption also activates Th1 cells, leading to intestinal mucosa inflammation [88]. Thus, intestinal barrier impairment due to untreated or undiagnosed CD can lead to the increased passage of pathogens, activating immune responses and increasing the risk of developing autoimmune diseases such as PSO [39].

Some recent studies have demonstrated a possible association between PSO severity or psoriatic arthritis and CD antibody positivity [89,90]. Woo et al. found in more than 100 PSO patients a significant association between elevated CD markers and increased PSO severity [89]. 

## 5. Potential Therapeutic Role of Nutrition in Psoriasis

### 5.1. The Role of Diet: A Protective Role toward the Most Serious Forms of Psoriasis

The well-known Mediterranean diet (MD) is characterized by the high consumption of whole grains, legumes, fish, vegetables, fruits, nuts, olive oil, and seeds; a regular controlled intake of dairy products like milk, yogurt, and cheese; and an occasional intake of red and processed meat [31]. Thus, the MD contributes to a high intake of monounsaturated fat from vegetable oils and a reduced intake of saturated fat from animal proteins such as meat or dairy products. The high quality of the lipid intake in the MD is associated with the regular consumption of fish rich in docosahexaenoic acid and eicosapentaenoic acid, nuts such as almonds, and extra virgin olive oil rich in oleic acid and vitamin E. On the contrary, the intake of red meat and processed meat, rich in saturated and trans fats, is reduced in the MD [31]. 

The MD is associated with a lower risk of non-communicable chronic diseases such as obesity, metabolic and cardiovascular diseases, neurodegenerative disorders, and cancers [31]. Recently, it was demonstrated that patients adhering to the MD are less likely to develop PSO than patients not following the MD [91]. The association between PSO severity and high body mass index (BMI), low physical activity levels, and high triglyceride levels confirmed the results of Phan et al. [92]. The results from a recent systematic review concluded that the MD is the gold standard nutritional approach for PSO patients [93]. The MD is also characterized by a reduced intake of sugars and increased consumption of carbohydrates such as whole pasta, whole bread, whole rice, and barley, which positively impact the levels of blood glucose and decrease insulin secretion, slowing down the synthesis of altered growth factors in the PSO patient such as epidermal growth factor (EGF) and insulin-like growth factor-1 (EGF-1) [94,95]. Other components of the MD like white meat, fish, eggs, and cheeses have an important role in maintaining lean body mass. The daily consumption of fresh fruits and vegetables rich in vitamins and polyphenols leads to antioxidant effects potentially beneficial in PSO patients [93].

Interesting data have emerged from calorie restriction. In particular, restricting calories lowers oxidative stress and the rate at which arachidonic acid is converted to leukotrienes. These mechanisms provide an explanation for why a low-calorie diet (LCD) is effective in treating PSO symptoms [96] An improvement in PASI could be achieved with cyclosporine treatment when a low-calorie diet was also prescribed, according to a randomized controlled trial on 61 obese patients [97]. Numerous clinical trials that demonstrated improvements in the PASI and Dermatology Life Quality Index (DLQI) supported the use of calorie restriction and the resulting weight loss as supplementary care for 290 obese or overweight patients [97,98,99,100]. The ability to adhere to a long-term restriction diet represented a significant barrier to patient outcomes because the calorie restriction in the studies was approximately 800–1000 kcal per day [96]. The management of psoriatic symptoms may benefit from an assessment by a nutritionist and the implementation of a tailored LCD to reduce fat mass [101].

Another interesting new potential role is emerging for GFD [102]. Indeed, a significant reduction in clinical PSO symptoms was observed in patients following GFD for a 3-month period [103]. Other case reports also demonstrated a complete reduction in symptoms after following GFD [104]. All these findings need to be confirmed with further studies in order to better define the mechanisms of the potential benefit of GFD on PSO and propose an adequate nutritional therapy [89].

### 5.2. Psoriasis and Dietary Supplementation of Fish Oil, Zinc, Selenium, Curcumin and Tryptophan

The potential therapeutic benefits associated with taking supplements with fish oil, tryptophan, selenium, zinc, and curcumin have been the subject of numerous studies [96,105]. Fish oil, which is high in omega-3 fatty acids, has demonstrated the potential in improving the clinical outcomes of psoriasis (PSO) patients. Eicosapentaenoic acid (EPA) and docosahexaenoic acid (DHA) intakes at effective doses varied considerably, with an average estimated intake of 4 g/day and 2.6 g/day, respectively; however, the recent research agrees on the effectiveness of this dietary supplementation in treating PSO symptoms [106]. While adding fish oil to the diet has been linked to a reduction in the severity of PSO, the results are still inconsistent, with some studies showing beneficial outcomes and others exhibiting poor outcomes [107,108,109]. As a result, it is challenging to make firm recommendations. PSO symptoms have been linked to a fall in serum selenium levels, a vital element with antiproliferative and immune-regulating properties. Coenzyme Q10, vitamin E, and selenium supplements may have a beneficial impact on disease development [110]. In a different study, people with psoriasis (PSO) significantly benefited clinically when supplemented with selenium along with folic acid, magnesium, iron, zinc, copper, manganese, chromium, iodine, and vitamins A, D, E, K, C, and B [111]. The effectiveness of selenium and zinc supplementation, despite this, is disputed in the literature [112]. Additionally, curcumin’s potential therapeutic role has been investigated, due to its well-known antioxidant and anti-inflammatory properties, suggesting that it may have a beneficial impact on enhancing PSO skin lesions [96].

Moreover, topical treatment with vitamin D or its derivatives is a safe and effective treatment and represents the first-line option for mild to moderate PSO. A study analyzing the effect of oral supplementation mainly showed an improvement in PASI score after a median intervention of 6 months; nonetheless, a recent meta-analysis reported that a clear beneficial effect of oral vitamin D supplementation connot be demonstrated and larger-sample-size randomized controlled trials are needed to provide stronger conclusions [113]. 

## 6. Limitation and Perspective

As a narrative review, our study has some clear limits since it is not structured on a systematic search. Nonetheless, we looked to underline the most recent evidence and investigate new possible strategies in the field of nutrition management of PSO patients.

From our review, it appeared clear that excessive body weight negatively influenced psoriatic inflammation. Hyperglycemia and insulin resistance seem to have a central role in the stimulation of keratinocytes and fibroblasts, and, interestingly, the level of serum Hb1c showed a significant association with PASI. Nonetheless, larger studies are needed to confirm this association and an exciting area of research may be represented by the use of GLP-1 receptor agonists in PSO patients with DM or obesity.

The analysis of gut microbiota, undoubtedly, represents another area of great interest. Since it emerged that “dysbiosis” is a common feature in these patients, the induction of a state of “eubiosis” through appropriate diet, use of probiotics, or even fecal transplantation can be, in our opinion, of great benefit to disease control.

Another unclear topic is the potential benefit of the GFD. Some studies reported symptom improvements with the adoption of this exclusion diet. Nonetheless, the studied sample sizes are limited, and the results appear weak. 

From our perspective, the main important findings that can be drawn from this review is that a healthy diet, structured on the Mediterranean model, can be very helpful for PSO patients because it has anti-inflammatory properties, allows the maintaining of proper body weight, favors good glycemic control, and provides vitamins and antioxidants.

It is always useful to investigate micronutrient deficiencies (especially folic acid, zinc, selenium, vitamin E, and vitamin D) and provide adequate supplementation when needed.

## 7. Conclusions

PSO is a chronic autoimmune disease affecting the skin. Its treatment mainly depends on the extension, localization, and disease severity. Dietary manipulation may not be intended as the primary treatment for PSO, but it can synergistically promote successful treatment outcomes and reduce the incidence of life-changing comorbidities including DM and CVD. Given the scientific evidence of the decisive contribution of nutrition in PSO therapy, the role of the clinical nutritionist in the care and involvement of the patient is fundamental. Although it has not been demonstrated that the adoption of a correct diet excludes the risk of PSO onset, it is evident that an appropriate dietary habit based on the Mediterranean diet (a balanced diet rich in fruits and vegetables, with a greater intake of bluefish and a lower consumption of red meats and cured meats) improves its clinical expression and reduces the incidence of PSO comorbidities. The intake of some foods can, on the contrary, aggravate the disease or act as a trigger. Exercise may be a helpful strategy against PSO and can potentially improve the disease in patients who are also overweight.

## Figures and Tables

**Table 1:** Nutritional issues and their potential therapies in Psoriasis-affected patients.

Nutritional Disorders Associated with Psoriasis	Potential Nutritional Therapies	References
Obesity	Low-calorie diet (LCD) Physical activity	Alotaibi et al., 2018 [30]; Phan at al., 2018 [31]
Hypovitaminosis D	Oral Vit. D supplementation Sunlight exposure	Theodoridis et al., 2021 [32]; Brożyna et al., 2022 [33]
Gut microbiota dysbiosis	Lifestyle Body mass index (BMI) Physical activity Eating habits Probiotics and prebiotics?	Rinninella et al., 2019 [34,35]; Palombaro et al., 2020 [36]
Diabetes	Low index and glycemic load diet	Hao et al., 2021 [37]
Non-celiac gluten sensitivity (NCGS)	Gluten-free diet (GFD)	Woo et al., 2004 [38]; Addolorato et al., 2003 [39]
Micronutrients deficiencies	Oral supplementation?	Yousefzadeh et al., 2017 [37]; Smith et al., 2009 [37]
